# Implantable vision-enhancing devices and postoperative rehabilitation in advanced age-related macular degeneration

**DOI:** 10.1038/s41433-022-02179-z

**Published:** 2022-07-22

**Authors:** Andreas F. Borkenstein, Eva-Maria Borkenstein, Albert J. Augustin

**Affiliations:** 1 Borkenstein & Borkenstein, Private Practice at Privatklinik der Kreuzschwestern, 8010 Graz, Austria; 2 Department of Ophthalmology, Städtisches Klinikum Karlsruhe, 76133 Karlsruhe, Germany

**Keywords:** Medical research, Quality of life

## Abstract

Age-related macular degeneration (AMD) results in progressive vision loss that significantly impacts patients’ quality of life and ability to perform routine daily activities. Although pharmaceutical treatments for AMD are available and in clinical development, patients with late-stage AMD are relatively underserved. Specialized rehabilitation programs and external low-vision aids are available to support visual performance for those with advanced AMD; but intraocular vision-improving devices, including implantable miniature telescope (IMT) and intraocular lens (IOL) implants, offer advantages regarding head motion, vestibular ocular reflex development, and depth perception. IMT and IOL technologies are rapidly evolving, and many patients who could benefit from them remain unidentified. This review of recent literature summarizes available information on implantable devices for improving vision in patients with advanced AMD. Furthermore, it discusses recent attempts of developing the quality of life tests including activities of daily life and objective assessments. This may offer the ophthalmologist but also the patient a better possibility to detect changes or improvements before and after surgery. It is evident that surgery with new implants/devices is no longer the challenge, but rather the more complex management of patients before and after surgery as well as the correct selection of cases.

## Abstract (zh)

年龄相关性黄斑变性(AMD)导致患者渐进性视力丧失, 严重影响其生活质量和日常活动能力。尽管有针对AMD的治疗性药物且临床用药还在研发中, 但针对晚期AMD患者的药物治疗相对匮乏。专门的康复计划及外部助盲设备能够改善进展期AMD患者的视力, 但是眼内植入式的设备包括可植入微型望远镜 (IMT) 和眼内晶体 (IOL), 在头部运动、前庭眼反射的发育及深度觉感知方面更具优势。IMT和IOL技术发展迅速, 并且还有很多可能从中获益的患者。本文通过回顾近期文献总结了进展期AMD患者植入视觉提高设备的可用的信息。此外, 本文讨论了近期研发用于改善生活质量的测试, 包括日常生活活动 (ADL) 和客观评估。这也为眼科医生和患者更好地监测手术前后视力改变或提高提供了可能性。显然, 使用新植入物或设备的手术不再是挑战, 而手术前后患者更复杂的管理以及病例的正确选择更具有挑战性。

## Introduction

Age-related macular degeneration (AMD) includes both a dry or atrophic form and a wet or neovascular type [1]. The dry form, characterized by retinal pigment epithelium (RPE) dysfunction, photoreceptor loss, and retinal degeneration, terminates in geographic atrophy (GA) [1]. Neovascular AMD involves the emergence and growth of new blood vessels in the space between the RPE and Bruch’s membrane. The barrier function of the endothelial cells comprising these fragile vessels is impaired, and they leak fluids and may even hemorrhage, events that damage the macula and can lead to loss or dysfunction of photoreceptors, the RPE, and the choroidal complex [2–4]. Of all AMD, the dry form accounts for 85–90% and the wet form for 10–15%, and the subtypes may overlap in a given patient [1]. The damage in AMD is progressive and manifests as ongoing loss of central vision and eventual blindness [3, 4]. AMD is the third-leading cause of blindness worldwide after cataracts and glaucoma and is projected to affect about 300 million people by 2040 [3–5]. The progressive deterioration of visual function in AMD has a significant impact on patients’ quality of life and reduces their independence in performing daily activities. As AMD progresses, patients need visual rehabilitation to continue performing routine tasks that require the use of central vision, such as reading and driving—activities that involve multiple visual functions, including acuity, contrast sensitivity, and reading speed [6, 7]. Early and intermediate stages of wet AMD can be treated using medications, such as vascular endothelial growth factor (VEGF) inhibitors; and current clinical trials are also evaluating therapeutics that may slow the progression of GA [8, 9]. However, patients with late-stage AMD remain an underserved population.

Many approaches have been employed to improve visual performance for people suffering from advanced AMD; for example, patients can be trained to improve their use of residual vision through specialized and individualized rehabilitation programs [10]. Residual and low-vision aids including electronic or optical magnifiers, colored filters for contrast sensitivity, prism spectacles, or closed-circuit television also can help magnify central vision in certain contexts [6]. However, external aids have important limitations, including restricted visual fields, cosmetic drawbacks, and the need for continual motion of the head, leading to vestibular effects [6].

Intraocular vision-improving devices, such as implantable miniature telescopes (IMT) and intraocular lens (IOL) implants, may be superior to external aids for improving vision in patients with advanced AMD because they provide technology that is more intuitive with respect to head motion, vestibular ocular reflex adaptation, and monocular depth perception [4, 11, 12]. Both IOL and telescopic implant technologies for advanced AMD are rapidly evolving. Their utility for patients with low vision, including those with AMD, requires a balance between optimal magnification and ease of use in daily activities. Because many patients go unidentified as possible candidates who can benefit from these newer technologies, there is a great opportunity to generate increased awareness for patients with low vision and those who care for them. This review summarizes recent information on implantable devices that can be employed to improve vision in patients with advanced AMD.

## Implantable devices for vision improvement in advanced AMD

### The IOL for Visually Impaired People (IOL-VIP) System

**Fig. 1:**
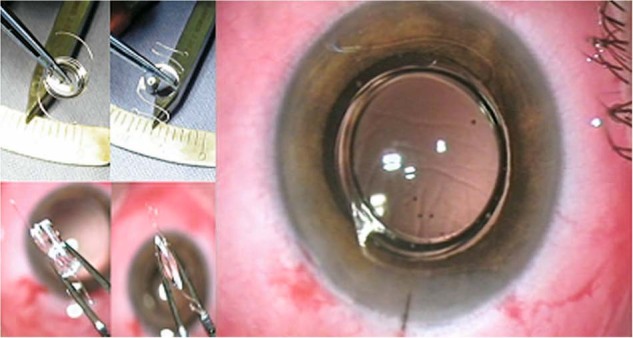
The IOL-VIP system. Front and side views of the in-the-bag (left) and anterior chamber (right) IOL-VIP and front view of the IOL-VIP system into the eye at the end of the surgical procedure [13].

The classic IOL-VIP system (Fig. 1 and Table 1) is a double IOL implant for the visual rehabilitation of patients with macular disease. It consists of a biconcave high-minus-power IOL in the capsular bag and a biconvex high-plus-power IOL in the anterior chamber, creating, together with the cornea, an intraocular Galilean telescope with ×1.3 magnification for distance [13].In a group of 40 eyes of 35 patients, this lens system was reported to be effective and well tolerated. It improved best-corrected visual acuity (BCVA), reading magnification, and reading distance [13]. The software provided with the IOL-VIP system can be used to estimate preferred retinal locus (PRL), reading speed, contrast sensitivity, and visual acuity [4]. It can also be used for training the PRL pre- and postoperatively and can enable the detection of >66% of patients whose PRL may be too far from the fovea and/or who are not responding adequately to pre-surgical training, decreasing the risk for implant removal [4]. However, fixation and focusing of the device on one PRL during the course of training may limit future performance as the disease progresses and PRL changes.

**Table 1:** Summary of lenses reviewed.

Device	Source	Technology type	Magnification	Lens status	Incision size (mm)	Monocular/ binocular	Rehab required	PRL selection required	Number of patients enrolled	Mean age	Gender	Race	Baseline BCDVA (logMAR) mean	BCDVA improvement (logMAR) mean	Baseline BCNVA (logMAR) mean	BCNVA improvement (logMAR) mean	Endothelial cell loss	Effect on QoL	VA test method
IMT	[19, 29]	Galilean Telescope	×2.2 and ×2.7	Phakic	10-11	Monocular	Yes	No	217	75.6	103 (47.5%) female 114 (52.5%) male	208 (95.9%) white 3 (1.4%) black 5 (2.3%) Hispanic 1 (0.5%) Asian	1.20	−0.35 (*p* < 0.0001)^a^	1.10	−0.32 (*p* < 0.0001)^a^	25–40%	Improved	BCDVA was measured by ETDRS. BCNVA was measured at 20 and 40 cm with the New ETDRS chart 1, using M-unit equivalents for each line of acuity measured. The better of the two BCNVA was reported
SING IMT	Data on file, [47]	Galilean Telescope	×2.7	Phakic	6.5	Monocular	Yes	No	3	75.6	3 (100%) male	NR	NR	NR	NR	NR	7%	NR	NR
LMI	[18, 19]	Cassegrain Lens	×2.5	Phakic	6.5	Binocular	Yes	No	6	NR	1 (16.67%) female 5 (83.33%) male	NR	1.47	−0.37 (*p* = 0.14)^a^	1.22^a^	−1.02 (*p* = 0.14)^a^	6%	N.S. (*p* = 0.14)	BCDVA was measured with a Snellen chart. BCNVA was measured using the ETDRS near vision chart at 20 cm
SML	[19, 21]	IOL	×2.0	Phakic/Pseudophakic	2.2	Monocular	No	No	8	NR	NR	NR	0.62^b^	NS	0.75^b^ at 40 cm 0.52^b^ at 15 cm	−0.21 to −0.44 at 15 cm (*p* = UNK)^a^	NR	NR	Radner chart in German was used to test reading vision
LENTIS MAX	[23]	IOL	×1.5 (N) to ×3 (D)	Phakic	2.2	Binocular	NR	NR	11	80.6	8 (72.7%) female 3 (27.2%) male	NR	0.69	−0.31 (*p* = UNK)	1.06	−0.38 (*p* = UNK)	NR	Improved	NR
IOL-AMD	[15, 19]	Double Intraocular Lens	×1.3	Phakic/Pseudophakic	2.8	Binocular	Yes	Yes	12	77	NR	NR	0.92^b^	−0.22 (*p* = UNK)^b^	0.85^b^	−0.18 (*p* = UNK)^b^	18%	NR	CDVA was measured with a Snellen chart. Near acuity was measured with N-point at 40 cm
IOL-VIP System	[13, 19]	Double Intraocular Lens	×1.3 (distance)	Phakic/Pseudophakic	7	Binocular	Yes	Yes	35	73	19 (54.3%) female 16 (45.7%) male	NR	1.29	−0.52 (*p* = UNK)	NR	NR	7–10%	NR	BCVA was evaluated by the ETDRS chart
EyeMax Mono	[17, 20]	IOL	×1.1–×1.2	Phakic/Pseudophakic	2.2	Binocular	No	No	244	80	146 (59.8%) female 98 (40.2%) male	NR	1.06	−0.35 (*p* < 0.0001)	1.36	−0.48 (*p* < 0.0001)	~7% (interpolated from the figure)	Improved	Full subjective refraction BCVA was measured. Near acuity was measured with N-point at 40 cm
Orilens	[19]	Cassegrain Lens	×2.5	NR	5.5	NR	NR	NR	NR	NR	NR	NR	NR	NR	NR	NR	NR	NR	NR

*BCNVA* best-corrected near vision acuity, *BCDVA* best-corrected distance vision acuity, *ETDRS* Early Treatment Diabetic Retinopathy Study, *logMAR* Logarithm of the Minimum Angle of Resolution, *NR* not reported, *NS* not significant, *PRL* preferred lens focus, *UNK* unknown.

^a^LogMAR calculated from ETDRS score based on methods outlined in Beck et al. (2003).

^b^LogMAR calculated from corrected distance visual acuity (CDVA) and corrected near visual acuity (CNVA) in Snellen decimal scale based on methods outlined in Mataftsi et al. (2019).

Clinical results for the IOL-VIP system indicate that it is well tolerated and does not interfere with peripheral or binocular vision [13]. This system also has significant limitations that include a need for perfect alignment between the two IOLs and the need for a relatively large (up to 7 mm) corneal incision for insertion. Adverse events associated with the IOL-VIP system include transient elevations in intraocular pressure (IOP), corneal edema, ocular pain, posterior capsule opacification, pupillary block, and anterior capsule fibrosis [4, 12–14]. The large incision may result in induced astigmatism and challenges with wound healing in the postoperative period. Other potential limitations for this system include a possible crowding effect with two lenses, particularly with one IOL in the anterior chamber that may increase the risk for glaucoma or angle closure, especially in patients with hyperopia [12]. Furthermore, the magnification is limited to ×1.3, and long periods of pre- and postoperative adaptation are required for the IOL-VIP, which may not be acceptable for some patients [12].

### IOL-AMD

**Fig. 2:**
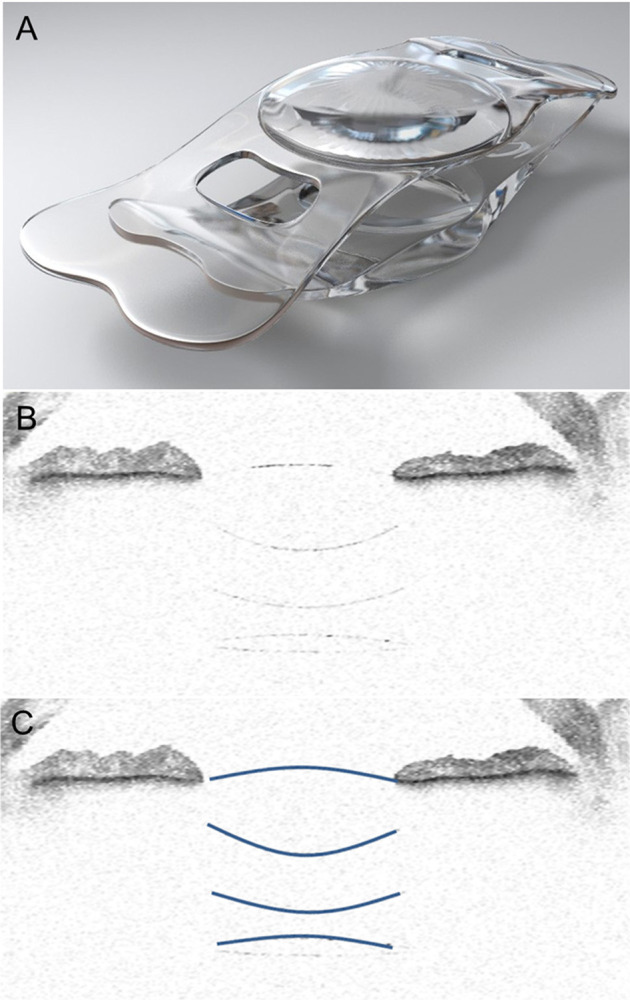
The IOL-AMD. Artistic rendering of the injectable telescopic IOL (**A**) and its appearance on anterior segment optical coherence tomography after implantation (**B**) with optic surfaces highlighted (**C**) [15].

The IOL-AMD (Fig. 2 and Table 1) uses the principle of the Galilean telescope (with the cornea) to produce ×1.25–×1.3 magnification with a visual field reduction of about 30% [15]. After the removal of the crystalline lens or existing IOL, one high-negative and one high-positive soft hydrophobic IOLs are injected individually into the capsular bag and ciliary sulcus, respectively, using 3-mm corneal incisions [15]. Results from 18 eyes of 12 patients indicated no significant intra- or postoperative complications and improvement in mean decimal corrected distance visual acuity (CDVA) from 0.12 preoperatively to 0.20 at 4 months. The mean change in spherical equivalent was 1.5 dioptres (D) with 0.5 D of induced astigmatism. Microperimetric testing in a subset of three patients indicated a magnification effect and a deviation of the retinal image by up to 5 degrees, with improved fixation stability [15]. Complications associated with this device included IOP elevation and anterior vaulting of the IOL in the capsular bag in one patient, which resulted in a decrease in visual quality [4]. An important advantage of this lens is a uniform breadth of focus across the macula because of traverse asphericity [4]. However, this approach also has some limitations, including a magnification that extends only to ×1.3. Moreover, further progression of AMD may require additional surgery due to the associated change in PRL [4]. Importantly, the normal range of daily activities typically requires multiple PRLs, and limiting the PRL to one area could cause further visual dysfunction. Because of these limitations, the manufacturing of the device has been discontinued [4].

### EyeMax Mono

**Fig. 3:**
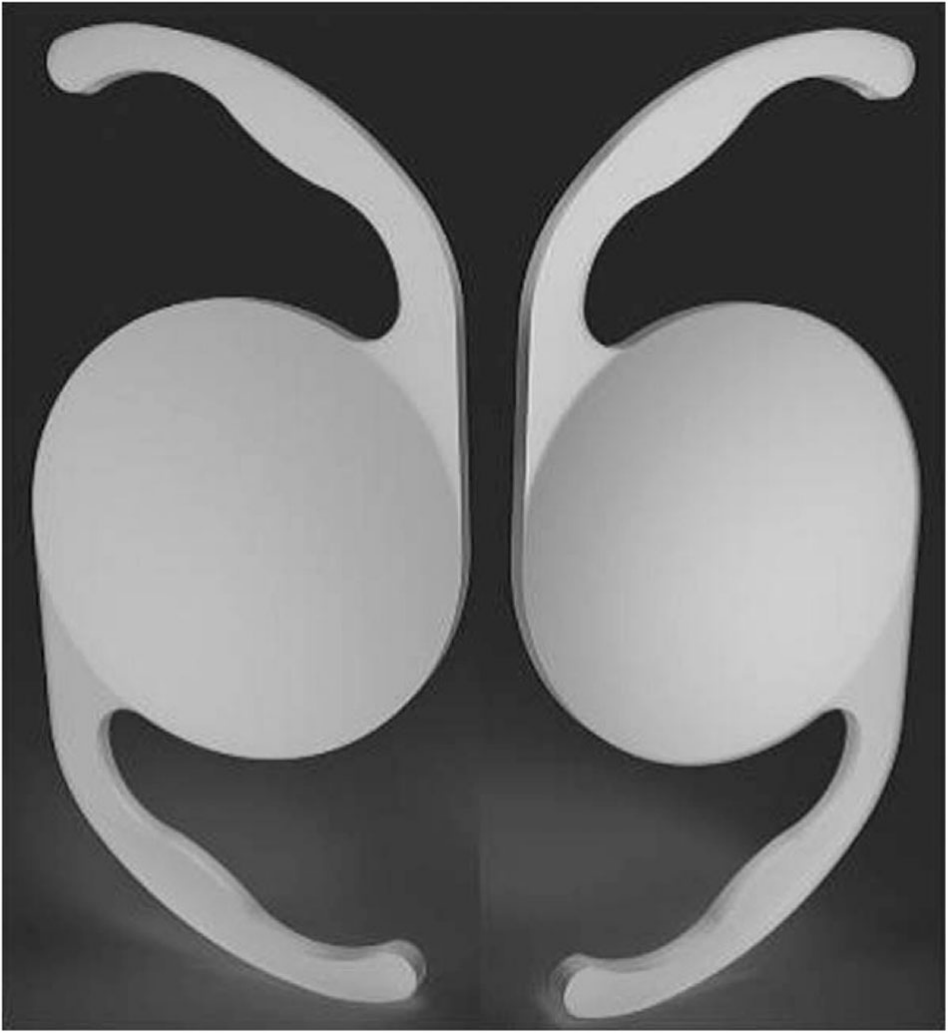
The EyeMax Mono [46]. It is a single-piece, hydrophobic acrylic IOL with an overall diameter of 13 mm.

This is a single-piece, soft, hydrophobic acrylic IOL, comparable to a standard IOL in terms of dimensions (6–13 mm) (Fig. 3 and Table 1). It improves image quality across the entire macula, increasing the breadth of focus and reducing blur. The optics of this lens are wavefront optimized with the aim of providing improved image quality for an area extending about 10 degrees from the center of the fovea [16]. It permits patients with single or multiple PRLs to gain optimum benefit from the most functional areas of their macula [17] and provides magnification from ×1.1 to ×1.2 [4]. EyeMax Mono is available in two versions: the first is engineered for capsular bag implantation following phacoemulsification, and the second is employed for sulcus implantation and use in combination with a previously implanted monofocal IOL [4]. Results from a consecutive case series of 244 eyes with dry or stable wet AMD and logMAR visual acuity ≥0.3 indicated a mean CDVA (logMAR) improvement from 1.06 preoperatively to 0.71 postoperatively [17]. Mean preoperative corrected near visual acuity (CNVA, logMAR) increased from 1.36 to 0.88 [17]. Complications associated with the implantation of the EyeMax Mono included anterior capsular tear, postoperative subretinal fluid, and elevated IOP [17]. As other authors have acknowledged, more information is needed about the efficacy, safety, and functional outcomes achieved with this lens [4].

### Mirror implants

**Fig. 4:**
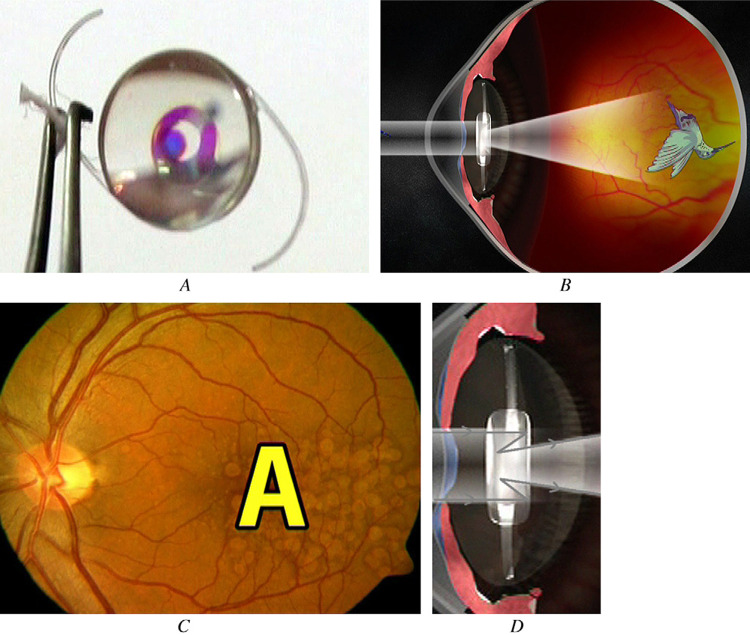
The Lipschitz macular implant (LMI). The LMI mirror telescopic IOL (**A**); illustration depicting how the LMI functions (**B**); the LMI magnifies the central image on the retina (**C**); and gray trace of light demonstrating the magnification caused by the mirrors (**D**) [18].

The first Lipschitz macular implant (LMI) (Fig. 4 and Table 1) was an IOL that used the principle of the Cassegrain mirror reflecting telescope [11, 18, 19]. Dielectric coatings on the LMI act as mirrors to produce a ×2.5 magnified image centrally on the retina and a regular-sized image in the periphery [18]. Results from six eyes of six patients (four with AMD and one each with myopic macular degeneration or macular dystrophy) indicated a mean gain in distance acuity of 3.66 lines and a mean increase in the Early Treatment Diabetic Retinopathy Study (ETDRS) score for near acuity of 50.83 logMAR [18].

Advantages of the LMI include the provision of ×2.5 magnification and the fact that a newer version of this device can be directly implanted in the sulcus (LMI-SI) [11]. Limitations associated with this lens include the fact that the LMI-SI, which is a non-foldable, one-piece IOL, requires enlarging incisions to as much as 5.5 mm [11]. In addition, all patients implanted with this lens experienced glare postoperatively, and two patients complained of shadowing which resolved by 3 months [18, 19].

### Bulb miniature lenses

**Fig. 5:**
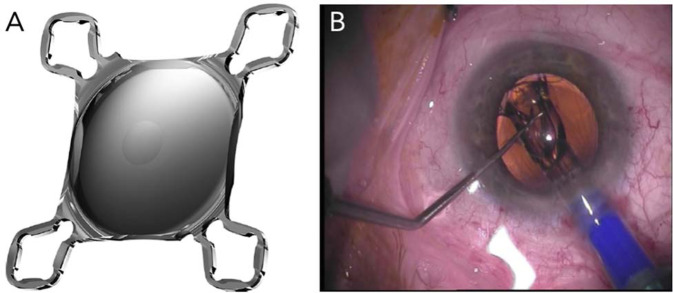
The Scharioth Macular IOL. Image of macular add-on IOL (**A**); intraoperative view during implantation of macular add-on IOL (**B**). The IOL is unfolding while an instrument through the side-port incision is guiding the leading haptic into the ciliary sulcus [21].

The Scharioth Macula Lens (A45SML) is a single-piece lens developed for the visual rehabilitation of patients with advanced AMD (Fig. 5 and Table 1) [20]. It is a macular add-on IOL developed for ciliary sulcus implantation in pseudophakic eyes and can be implanted during uncomplicated standard phacoemulsification with in-the-bag posterior chamber IOL implantation, or years after cataract surgery [21]. The lens has a central portion of 1.5 mm diameter with addition of +10 D. The magnification is ~×2.0 for very near vision only when calculated mathematically, but in practice depends on both the anatomy of the eye and the final reading distance. The overall diameter of the IOL is 13.0 mm with four symmetric haptics [21].

Results from a prospective multicenter trial that included 50 eyes of 50 pseudophakic patients with either dry or previously treated wet AMD that was stable for ≥6 months showed a mean CNVA improvement from 0.23 preoperatively to 0.57 at 1 year postoperatively. The mean preoperative CDVA was 0.19, which did not change postoperatively. One patient had the lens explanted 3 months postoperatively due to glare/halos [22].

This lens has multiple advantages. It is designed to enhance near vision only with reduced reading distance and maximum magnification, without affecting or enhancing peripheral vision [11, 19]. It is also one of the few lenses that can be implanted as part of routine cataract surgery as well as in pseudophakic patients, and only a small incision (2.2 mm) is required for implantation [11, 21]. Limitations of the A45SML include the fact that it is contraindicated in patients with other eye conditions including chronic uveitis, zonular weakness, secondary cataracts, and central corneal opacities [11]. Notably, magnification of objects is possible only when they are within 10–15 cm of the eye [11].

### Magnification IOLs

**Fig. 6:**
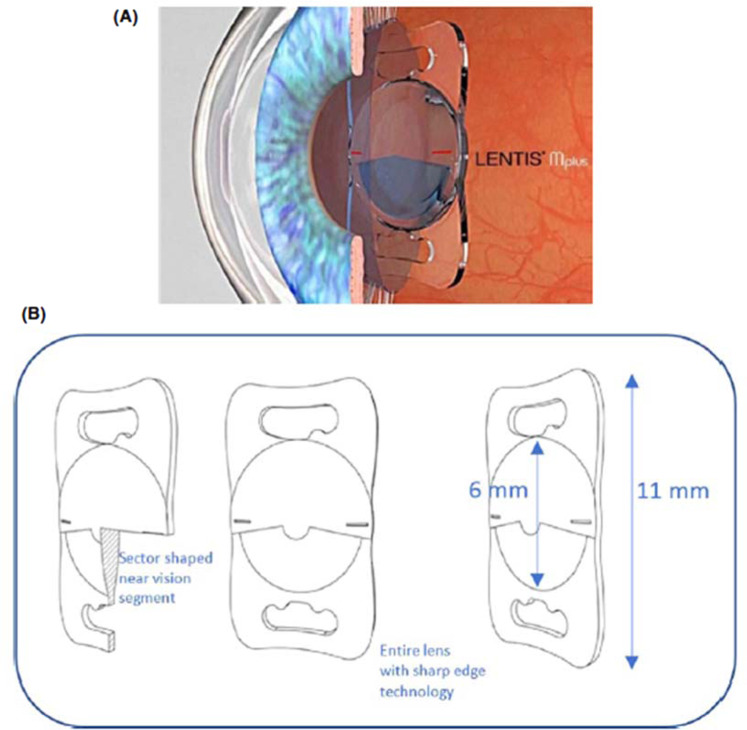
The Lentis MAX. Sketch of the Lentis LS‐313 MF80 (**A**); and specifications of Lentis LS‐313 MF80 with sector‐shaped near vision segment and sharp edges (optic and haptic) (**B**) [25].

LENTIS MAX is a monofocal, hydrophobic, acrylic, aspheric IOL that enables a ×3 magnification at a distance of 15 cm [23, 24]. This biconvex lens with the aspherical surface that has two sectors with a total additional power of +8 dioptres [25] (Fig. 6). It has been employed for magnifying cataract surgery (MAGS) in 15 patients with advanced dry AMD. Eleven of these patients were followed for up to 48 months and all reported functional gains in the first 3–6 months after surgery. In addition, 10 of the 11 patients reported improved quality of life [23]. Other benefits include a routine procedure that does not introduce additional risks, as the lens has standard dimensions. These lenses are not available at present due to a calcification-related recall of another lens produced by the company [26].

### Implantable miniature telescope prosthesis

**Fig. 7:**
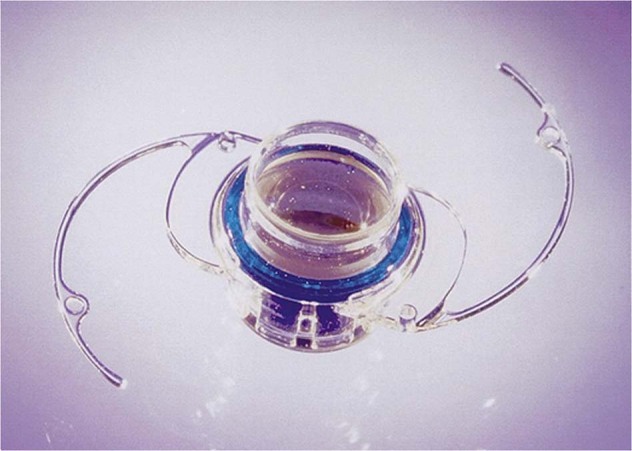
The Impantable Miniature Telescope (IMT). The IMT (view of the anterior aspect) is 4.4 mm long and 3.6 mm in diameter and weighs 115 mg in air. The central glass optical cylinder of this visual prosthetic device houses high-plus and high-minus micro-lenses. The optic is centered in a clear polymethylmethacrylate (PMMA) carrier plate with modified C-loops. The blue PMMA ring serves as a light restrictor, designed to prevent glare [28].

The IMT was invented by Isaac Lipschitz and is based on the principle of fixed-focus Galilean telescopes [11, 19]. The IMT is designed from ultraprecision quartz glass and wide-angle micro-optics (Fig. 7 and Table 1) [27]. Together with the cornea, the IMT telephoto effect enlarges objects in the central visual field [27]. Because the device is implanted only in one eye, peripheral vision is compensated by the fellow eye [11, 27]. The IMT is available in two wide-angle magnifications (×2.2 and ×2.7) and requires approximately 10- to 11-mm incision for implantation [19, 28, 29]. It was first evaluated in a phase 1 trial that included 14 patients ≥60 years of age with bilateral GA or disciform scar AMD and cataract. At 12 months, 77% of 13 patients gained ≥2 lines of either distance or near BCVA, and 62% gained ≥3 lines; scores for activities of daily living (ADLs) improved for all patients [28].

The efficacy and safety of the IMT have been confirmed in a 1-year study with an additional 1 year of follow-up that included 217 patients with AMD and moderate-to-profound bilateral central visual acuity loss resulting from GA, disciform scar, or both [29, 30]. At 2 years, 59.5% of 173 telescope-implanted eyes gained ≥3 lines of BCVA compared with 10.3% of 174 fellow eyes [30]. Mean BCVA improved by 3.6 lines and 2.8 lines from baseline in eyes with the ×3 (nominally ×2.7) and ×2.2 lenses, respectively. Most patients also had sustained improvements in the ability to carry out ADLs [30]. Five-year follow-up of these patients indicated retention or improvement in best CDVA and corneal endothelial cell density (ECD) loss consistent with that reported for conventional IOLs [27]. This lens has been approved by the United States Food and Drug Administration (FDA) for implantation in patients ≥65 years who have a natural lens in at least one eye and who meet other criteria for health and overall vision [31]. It has also received the Conformité Européenne mark for the treatment of end-stage AMD [11]. Moreover, it is worth noting that treatment with this lens has been shown to be cost-effective, with a very low cost per quality of life-year gained [32].

It is worth emphasizing that the placement of IMT does not interfere with standard monitoring (e.g., with ocular coherence tomography) [33]; or with adjunctive treatments such as administration of intravitreal injections [33], laser photocoagulation [34], laser-assisted cataract surgery [35] or pars plana posterior capsulotomy [36].

### The Smaller-Incision New-Generation IMT

**Fig. 8:**
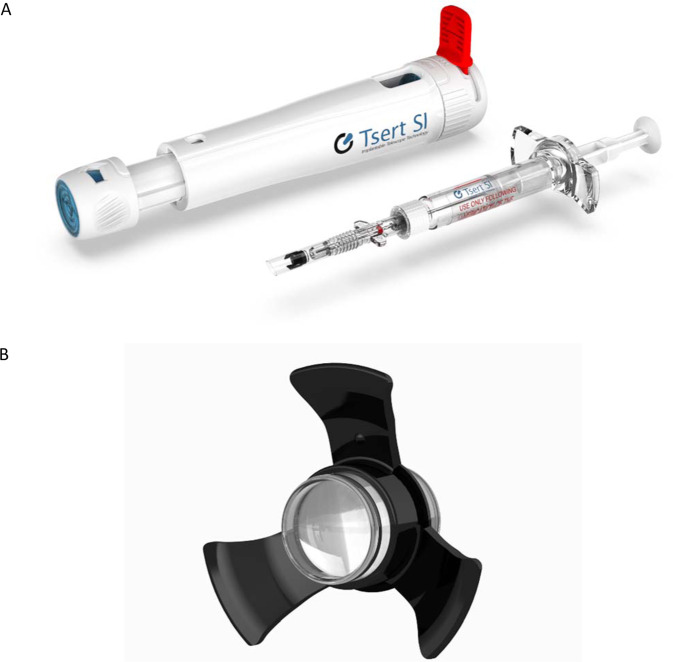
The SING IMT and its delivery system. Tsert SI Injector (**A**) and SING IMT Implant (**B**) (provided by Samsara Vision, Inc).

The Smaller-Incision New-Generation IMT (SING IMT) (Fig. 8 and Table 1) is a newer version of IMT designed with a pre-loaded delivery system. It requires a 6.5-mm incision, and surgery time is less than 30 min [37]. The smaller incision size with the SING IMT also significantly reduces surgical trauma, induced astigmatism, the number of sutures required, and loss of ECD, which permits more rapid initiation of rehabilitation [4, 37]. Both IMT and SING IMT have similar magnification ranges (×2.2 and ×2.7 nominal, respectively) [4] and aid vision at near, mid, and far-range distances. Other similarities and differences between the IMT and SING IMT are summarized in Table 2 [4].

**Table 2:** Comparison between the IMT and newer SING IMT.

Parameters	IMT	SING IMT
Magnification	×2.2/×2.7	×2.7
Optics diameter (mm)	3.60	Same
AXL (height, mm)	4.40	Same
Overall diameter (mm)^a^	13.5	10.8
Incision size (mm)	10–12	6.5–7.5
Capsulorhexis size (mm)	7	5.5
ECD loss	23–25%	7.9%
Corneal clearance post op ACD (mm)	2.5	3.5
Sutures	8–12	3–4
Manipulation	High rate	Almost none
Surgical duration (min)	60	<30
Surgical procedure	For skilled surgeon	Simpler, less complicated

Adapted from Grzybowski et al. 2020.

*AXL* axial length, *ECD* endothelium cell density, *ACD* anterior chamber depth.

^a^Including the haptic loop.

## Late AMD patient management

The devices reviewed in this paper are typically reserved for patients with late AMD and are employed when medical treatments such as anti-VEGF antibodies are no longer able to maintain adequate vision [38]. These devices provide the greatest benefit when they are combined with training in their use, as well as low-vision rehabilitation [39]. It is important to emphasize this fact to patients and to understand that achieving the greatest benefit from low-vision devices requires the collaboration of the patient with multiple healthcare professionals, including ophthalmologists, occupational therapists, psychologists, and social workers [38, 39]. Patients’ understanding and commitment to these interventions are essential [40]. For practitioners, it is important to focus more on patients’ ability to conduct ADLs that are important to them vs their performance on standardized visual acuity charts [16].

Indeed, a novel instrument, the “ADL-test kit” is being developed to pre- and postoperatively assess patients with AMD who are undergoing cataract surgery. This series of evaluations (a patient-reported questionnaire, a psychosocial/depression screening test, and a cognitive test) is intended to help ensure that recommendations about implantable devices and rehabilitation strategies line up with patients’ abilities, goals, and expectations [41]. These combined efforts have the potential to slow the progression of vision deterioration and preserve independence and quality of life [38, 42].

An important consideration for both the IMT and SING IMT is the requirement for substantial visual rehabilitation for patients to become accustomed to these lenses for static vision, navigation, and depth perception [11, 19]. In the pivotal 1-year trial of the IMT, patients were asked to participate in six visual rehabilitation visits to learn how to use their new lenses, including practice in alternating viewing between eyes for peripheral and central visual tasks [30]. One study reported that this training may take from 3 to 6 months in some patients [11], and another suggests that for other patients, rehabilitation may continue for as long as 6 months to 1 year [4]. Thus, a fairly high level of patient motivation and commitment to rehabilitation is required, in addition to generally good cognition, understanding of the technology, realistic goals, and a support network [16]. Training and rehabilitation exercises affect neuroadaptation, and a close relationship between a patient and their physician, who can guide them appropriately, will help to achieve the best results.

## Discussion

The results summarized here indicate a wide range of intraocular implants for patients with advanced AMD. These lenses can be compared based on features (Table 1). However, it remains difficult to make comparisons of efficacy or safety in the absence of head-to-head comparative studies. For several of the reviewed lenses, interpretation and generalization of clinical results is limited by both the very small number of patients evaluated, as well as short follow-up periods in clinical studies. One exception to this generalization is the IMT, which was evaluated in over 200 patients [29] and for which there are now 5 years of follow-up data [27]. The IMT is also the first and only FDA-approved implantable medical device demonstrated to improve vision and quality of life in qualified individuals with advanced AMD [12, 19]. Long-term corneal endothelial cell loss, a potential concern regarding the IMT, appears to be comparable to rates in patients undergoing routine cataract surgery; according to a 4-year follow-up of patients completing the 1-year IMT trial, 4 of 217 patients required a corneal transplant, and the rate of ECD loss was 3% per year [12, 27]. According to another report, ECD loss was substantially reduced with the SING IMT vs the original IMT device [4]. Concerns about the size of the original IMT implant prompted the development of the SING IMT, which is expected to decrease ECD loss. It was certified for use in Europe in 2020 and plans for evaluation in a clinical trial in the United States are currently underway [37].

Additional important considerations in the evaluation and comparison of intraocular devices for patients with advanced AMD are the influences of patient selection, pre- and postoperative management, and appropriateness of study endpoints. Patients most suited for IMT are ≥65 years of age with VA ranging between 20/160 and 20/800. These patients have bilateral central scotomas associated with end-stage AMD, disciform scar or GA, and cataract. They are also required to show an improvement of ≥5 letters with external visual aids in preoperative tests [31]. Such detailed guidance is important for any product used in this setting [16]. The experience and skills of the ocular surgeon and that of the rehabilitation specialist are likewise key determinants of the clinical outcomes achieved with these lenses [11, 16, 19]. It has also been noted that chart-based assessments of vision, such as the ETDRS, may not be the most appropriate technique to evaluate baseline visual impairment and treatment outcomes in patients with advanced AMD; and assessments focused on function and ADL may be more relevant [16]. This limitation applies to the testing of many lenses included in this review, for which clinical studies did not include an assessment of their effects on patients’ quality of life or ability to carry out ADLs (Table 1). Here again, the IMT is an exception to this generalization. Results at 1 year of follow-up among patients who received this lens indicated statistically and clinically significant (≥5 points) improvements from baseline in seven of the eight relevant domains of the National Eye Institute 25-item Visual Function Questionnaire that were correlated with improvements in BCVA [29].

Results for the Activities of Daily Life Questionnaire also indicated significant improvements for distance, intermediate, and near activities for both static and dynamic dimensions [29]. Importantly, there are still unmet needs for a validated test of visual function, or a patient-reported outcome measure developed specifically for individuals with AMD [16]. The authors are currently involved in developing a separate regimen precisely for this group of patients. Indeed, the primary challenge with these devices is not the surgery and implantation procedure, but rather the correct selection and management of patients.

The MACUSTAR program is also trying to address this problem and has the goal of developing new functional, structural, and patient-reported outcome measures for patients with intermediate AMD [43]. However, it is not clear whether any of these measures will also be validated in patients with end-stage disease.

A progressively debilitating disease like AMD requires progressive, individualized optical solutions. Devices with lower magnification (×1.2–×1.3) can help patients with low vision (20/160 to 20/240), whereas those with higher magnification (up to ×3) can help wider groups of patients with progressive AMD, having up to 20/800 VA. An additional advantage of devices with a larger magnification range, such as the IMT, is that they may be able to help patients with advanced AMD through a longer course of their disease progression.

While the optical-based devices for low vision considered in this review have all been demonstrated to improve vision for patients with later-stage AMD, they may not be sufficient for patients with very severe disease and advanced retinal degeneration. A few devices have the potential for the treatment of patients with very limited or no residual retinal function (i.e., those with visual acuity of 20/1200 or worse).

One such device is the Argus II, comprised of a chip containing an electrode array that is implanted on the surface of the retina and that stimulates retinal ganglion cells in response to wireless input from a camera mounted on a pair of glasses [44, 45]. Other implants in development are placed beneath the retina and are aimed at stimulating photoreceptors [39, 40, 44, 45]. These systems are only effective when sufficient retinal cells are present to initiate signaling to the brain. An alternative approach being developed for conditions in which this is not the case (e.g., advanced diabetic retinopathy or glaucoma) is to use the output of a camera to directly stimulate cells in the primary visual cortex [44, 45]. Many other approaches, including electrical stimulation of cells in the lateral geniculate nucleus, magnetic stimulation, and nanoparticle-based stimulation, are also in development [44]. Notably, time is an important factor in device development, in addition to the cost. To make these devices accessible to a wide range of people, well-defined protocols for surgical procedure and postoperative care must be established.

In conclusion, there are multiple intraocular vision-improving devices available for intervention in patients with advanced AMD. While these advances in technology can offer hope to many patients, it is difficult to predict how well the results in the clinical literature will generalize to actual practice. Only the IMT is FDA-approved for the treatment of these patients, and it is the only lens supported by results from a large-scale, prospective, long-term clinical trial [46]. Data for most other options have been derived from case studies or series with short-term follow-up [4, 46]. The decision regarding lens selection should only be made after careful discussion with the patient, whose commitment is essential for the successful completion of the rehabilitation process.

## Literature search

A literature search was conducted using the PubMed and Scopus search engines (https://scopus.com and https://pubmed.ncbi.nlm.nih.gov/) for the period between January 1950 and January 2022. The following words were searched in various combinations or as standalone: age-related macular degeneration (ARMD); intraocular vision-improving devices; implantable vision-improving devices; vision rehabilitation; low-vision aids; improving quality of life in ARMD; cataract surgery and intraocular lenses in ARMD; implantable miniature telescopes; improving vision in ARMD; developing quality of life tests in ARMD; activities of daily life (ADL) in ARMD; ADL tests; objective assessments in ARMD cases; activities of daily life questionnaire; functional, structural, and patient-reported outcome measures in ARMD cases. Publications were critically appraised, and relevant information was included in this review and cited accordingly. In addition, web pages of the manufacturers of such devices and various marketing materials were critically reviewed. The authors want to emphasize that the literature search was taken very seriously and was controlled and reviewed by all authors here, although such an article can never guarantee 100% completeness. New results and data from newer studies after completion of this review article can change the factual situation. This review tries to give an objective overview of the whole subject.

## Data Availability

The data that support the findings of this review article are available from the corresponding author upon reasonable request.
